# An Agent Independent Axis for Executed and Modeled Choice in Medial Prefrontal Cortex

**DOI:** 10.1016/j.neuron.2012.07.023

**Published:** 2012-09-20

**Authors:** Antoinette Nicolle, Miriam C. Klein-Flügge, Laurence T. Hunt, Ivo Vlaev, Raymond J. Dolan, Timothy E.J. Behrens

**Affiliations:** 1 Wellcome Trust Centre for Neuroimaging, University College London, 12 Queen Square, London WC1N 3BG, UK; 2 Department of Psychology, University of Hull, Cottingham Road, Hull HU6 7RX, UK; 3 Sobell Department of Motor Neuroscience and Movement Disorders, University College London, London WC1N 3BG, UK; 4 Centre for Functional MRI of the Brain, University of Oxford, John Radcliffe Hospital, Oxford OX3 9DU, UK; 5 Centre for Health Policy and Department of Surgery and Cancer, Imperial College London, St. Mary’s Campus, London W2 1NY, UK

## Abstract

Adaptive success in social animals depends on an ability to infer the likely actions of others. Little is known about the neural computations that underlie this capacity. Here, we show that the brain models the values and choices of others even when these values are currently irrelevant. These modeled choices use the same computations that underlie our own choices, but are resolved in a distinct neighboring medial prefrontal brain region. Crucially, however, when subjects choose on behalf of a partner instead of themselves, these regions exchange their functional roles. Hence, regions that represented values of the subject’s executed choices now represent the values of choices executed on behalf of the partner, and those that previously modeled the partner now model the subject. These data tie together neural computations underlying self-referential and social inference, and in so doing establish a new functional axis characterizing the medial wall of prefrontal cortex.

## Highlights

► Valuation and choice for self and other exhibit parallel computations in PFC ► vmPFC computes choices that will be executed, whether for self or other ► Rostral dmPFC computes choices that are modeled, whether for self or other ► A similar gradient is present in temporoparietal cortex

## Teaser

Nicolle et al. show that valuation and choice for self and other exhibit parallel computations, where gradients exist within both medial prefrontal and temporoparietal cortices. Ventral regions compute choices that will be executed, while dorsal regions compute choices that are merely modeled.

## Introduction

Across many species, behavior is driven by an ability to evaluate candidate actions with respect to one’s own motives. In social animals, the success of different actions may also depend on the preferences and actions of other individuals. Thus, adaptive success crucially depends on our ability to make inferences about the motives, values, and likely actions of others. In human behavior, this social valuation process is often a dominant factor shaping decisions. For example, a central factor determining the value of a new property is what the buyer believes other people will pay for it when it is later resold. When purchasing a new piece of clothing, the buyer (if not a neuroscientist) will often consider how it will appear in the eyes of others.

In the human brain, two neighboring regions of medial prefrontal cortex are often implicated in these two different functions (Amodio and Frith, 2006). In studies of decision making and neuroeconomics, ventromedial prefrontal cortex (vmPFC) consistently reflects an individual’s subjective valuation (Behrens et al., 2008; Boorman et al., 2009; Knutson et al., 2005; Plassmann et al., 2007). By contrast, its dorsomedial neighbor (rostral dmPFC) predominates in studies of social cognition when an individual must impute intentionality to others—exhibiting so-called theory of mind (Behrens et al., 2008; Frith and Frith, 1999, 2006; Hampton et al., 2008; Saxe, 2006).

While vmPFC responses to valuation and goal-directed choice are the subject of several computational theories (Boorman et al., 2009; Hare et al., 2011; Hunt et al., 2012; Levy and Glimcher, 2011; Lim et al., 2011), only scant attention has been given to computational mechanisms underlying dmPFC social responses (Behrens et al., 2008; Hampton et al., 2008; Yoshida et al., 2010). One possibility is that the ability to impute the intentions (Frith and Frith, 2006) and predict the actions (Behrens et al., 2008) of others derives from the same mechanisms that allow us to reflect on our own goals and actions (Amodio and Frith, 2006; Buckner and Carroll, 2007; Mitchell, 2009). Such a theory is appealing, as it would allow a computational understanding of goal-directed choice to be extended to social inferences.

However, this idea is difficult to reconcile with the existence of brain regions that appear specialized for processing self and other. Instead, it raises the intriguing possibility that a functional specialization in rostromedial prefrontal cortex (and in related temporoparietal cortex; Mitchell, 2008; Saxe and Wexler, 2005) is driven more by differences between choices that are executed versus those that are imagined or modeled. Teasing these two possible functional architectures apart is problematic as they are almost always aligned in cognitive tasks, where self-choices tend to be executed and others’ actions and intentions modeled.

Here, we describe neural signals that compute the choice preferences of another individual, whether or not they are relevant to the current choice. These signals precisely mirror well-studied signals reflecting personal choice preferences. Furthermore, by designing situations in which values of self and other may be either modeled or executed, we show that the functional gradient in the medial prefrontal cortex does not align with the individual, but is dependent on whether choices are executed by the subject or instead are modeled without overt execution.

## Results

**Figure 1:**
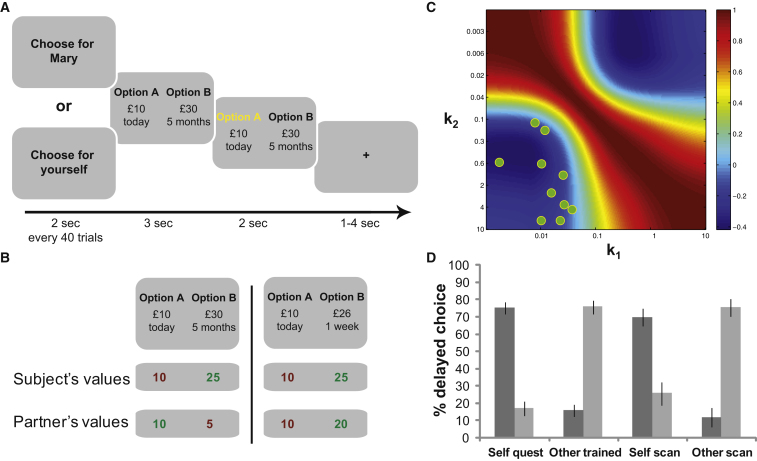
Experimental Design and Behavior (A) Trial timeline for the fMRI task of delegated intertemporal choice. In blocks of 40 trials, the frame of reference is changed between choosing for self and for partner. (B) Illustrative valuations from two example trials. Here, the subject exemplifies a relatively low discounter, as shown by the small influence of delay on value, while the partner is a relatively higher discounter. Note that there are four values to consider in the task: preferred and nonpreferred values for the subject and the partner. By computing value differences in the two frames of reference, we could dissociate both the different discount rates and the different choices of the two individuals. In this example, the subject’s value difference would be 15 in both trials, but the partner’s would be 5 and 10. (C) Correlation matrix between predicted value-related BOLD responses for partners with different temporal discount rates (*k*). Predicted response is assumed to align with the difference between chosen and unchosen values of each player. Reward and delays in the choice set were optimized to minimize overall predicted correlations (see Supplemental Information). Participants were prescreened to measure their discount rates and then paired to minimize correlations. Green dots represent the pairs of participants. Indeed the self and other value differences were broadly decorrelated in the experimental data (mean r = −0.11). (D) Average percent choice of the high-value long-delay option for high discounters (light) and low discounters (dark). Shown are choices in the prescreening questionnaire, during training on their partner’s choice preferences, and when choosing for self and for other in the fMRI experiment. Error bars show the standard error of the mean. Note that high discounters were paired with low discounters and vice versa. The behavioral flipping indicates that subjects learnt their partner’s preferences. See Figure S1.

To examine neural computations for self and others in medial prefrontal cortex, we designed a delegated intertemporal decision-making task (Figure 1A). Subjects chose between a large monetary reward delivered later, and a small reward delivered sooner. Critically, we asked subjects to choose for themselves in some trials, but in other trials to choose on the basis of what a partner participant would have chosen in the same context (Figure 1A). It is known that different individuals display significant variability in their preferences, with “low-discounters” preferring to wait for a later higher-value option and “high-discounters” preferring the more immediate smaller reward (Kable and Glimcher, 2007). This behavioral variability is quantified by estimating a subject’s unique “discount rate,” a parameter that captures an individual’s disposition to discount the value of delayed relative to more immediate reward.

We were interested in neural signals that distinguish the two individuals both in terms of their subjective valuations and in terms of their choices. In previous studies of value comparison, vmPFC activity has been found to correlate with the subjective value difference between chosen and unchosen options (Basten et al., 2010; Boorman et al., 2009; FitzGerald et al., 2009). Because this signal distinguishes between chosen and unchosen values, it is assumed that this region accesses both subjective values and choice (Wunderlich et al., 2010). In our delegated choice task, however, there are four different values to consider (Figure 1B). We reasoned that a signal that represents the subject’s own choices would correlate with the difference in valuations between the subject’s preferred and nonpreferred options (Basten et al., 2010; Boorman et al., 2009; FitzGerald et al., 2009). Similarly, a signal that represents the partner’s choices should also reflect a value difference signal, but here computed according both to the partner’s own values and choice preferences (i.e., the partner’s valuation of the option that the partner would have chosen minus the partner’s valuation of the option that the partner would have left unchosen) (Figure 1B).

Crucially, we required that these two value difference signals (self and other) could be identified simultaneously in evoked brain activity. We took two steps to ensure this would be the case (see Supplemental Information for more detail and Figure S1 available online). First, we prescreened 87 potential participants, using their choices in an online intertemporal choice questionnaire to estimate their individual discount rate. Twenty participants were then paired for the main experiment, such that each pair of individuals comprised one high and one low discounter. Consequently, by design, there would be many trials where the two partners express preference for different options (Figure 1B; Table S1). Second, we optimized the selection of intertemporal choices presented in the scanner such that the subjective value signals of the two participants (determined by their unique discount rates) would be maximally decorrelated (Figure 1C). An example of how this approach decorrelates the different choice variables can be found in the Supplemental Information.

Prior to scanning, ten pairs of subjects completed a trial-and-error learning session in which they each could learn their partner’s preferences from their online prescreen questionnaire choices (Figure 1D; see Supplemental Information). Partners then met each other and were subsequently each scanned, with their partner viewing from the operator room. During fMRI scanning, participants were presented with a new set of intertemporal choices in blocks of 40 trials. In each block, the subject in the scanner made choices either on behalf of themselves, or on behalf of their partner. At the end of the experiment, two of their actual choices would be realized: one prize randomly selected from the self-regarding blocks would go to the subject, and one from the other-regarding blocks would go to the partner.

We reasoned that, if the functional organization of medial frontal cortex is tied to the frame of reference of the individual (Behrens et al., 2009; Jenkins et al., 2008), then the vmPFC signal would always reflect the subject’s own value difference and the rostral dmPFC always reflect their partner’s value difference. In other words, the mPFC would show a functional gradient along an axis of self (ventrally) to other (dorsally). In contrast, if the organization is tied to the relevance of valuation for current choice, then this axis would show a gradient of executed values (i.e., self values during self choice and other values during other choice) to modeled values (i.e., other values during self choice and self values during other choice).

**Figure 2:**
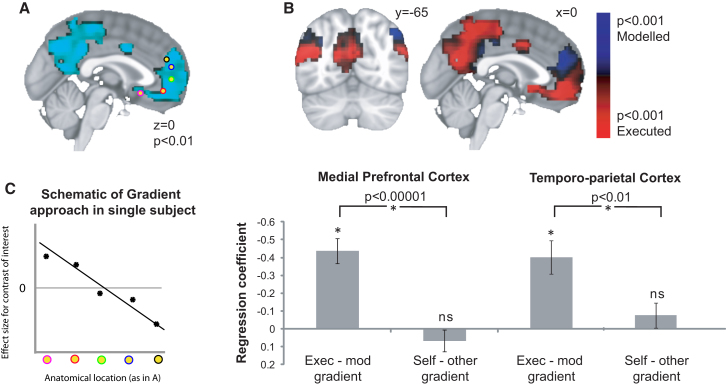
Gradient of Functional Organization in the Rostromedial Prefrontal Cortex (A) Regions of prefrontal cortex responding to the average contrast of value difference over all trials, i.e., across both self and other values (p < 0.01). Yellow dots are equally spaced along the activity profile and serve as the spatial markers for the analysis in (C). (B) Within the region of overall value sensitivity (shown in A), some regions respond more to the value difference that will be acted on in the current trial (executed in red), and some to the currently irrelevant value difference (modeled in blue). (C) Left: A spatial gradient analysis of functional contrast against position along the ventral-dorsal axis of medial prefrontal cortex (see colored dots in A). Left: In each subject, data from five anatomical locations are mapped onto a line and the spatial regression slope is computed. Right: Across subjects there is a strong gradient, with executed value effects expressed in more ventral and modeled value effects in more dorsal zones. No such dorsoventral gradient exists for the contrast of self-versus-other. The difference between the two gradients (indicating a difference of a difference) was significant (p < 0.00001). Error bars show the standard error of the mean. Right: Results of an equivalent spatial gradient analysis in the temporoparietal cortex. Sagittal sections through TPC are shown in Figure S2E.

To test these two opposing hypotheses, we recomputed subject’s discount rates and resultant valuations on the basis of the choices made in the scanner and identified regions of the brain that correlated with value difference averaged across both reference frames (Figure 2A), i.e., highlighting value-sensitive regions independently from their preferred frame. Within these regions, we tested whether there was a functional gradient along an axis of either self versus other, or executed versus modeled. We identified a large value-sensitive region spanning the medial wall of the rostral PFC (Figure 2A), which provided a functional mask reflecting any value difference encoding that was orthogonal to the statistical tests subsequently performed. Within this mask, no gradient was apparent when we compared self to partner value differences, but a clear ventral-dorsal gradient was immediately apparent when we compared executed to modeled value differences (Figure 2B), with more ventral regions reflecting executed and more dorsal regions modeled choices.

To perform a formal test of these differences, we fitted a regression slope to data extracted at five distinct locations spanning a ventral-dorsal axis (Figure 2A; Figure S2). Put simply, we tested whether there was a linear relationship between spatial position and functional coding. Across the group, we found a significant gradient along an executed/modeled axis (t[18] = 6.28, z = 4.513, p < 0.00001), but no such gradient for self versus other (t[18] = −1.06, z = −1.02 p > 0.30). The difference between these two gradients, indicative of the two candidate functional organizations, survived a formal comparison (paired t[18] = 6.18, z = 4.47, p < 0.00001; Figure 2C). We also note that, among other regions implicated in valuation, a similar gradient was exhibited in temporoparietal cortex (TPC) (x = −34 to −54, y = −54, z = 20 to 38, t[18] = 4.25, z = 3.49, p < 0.0005). Again, no such gradient could be found when we analyzed the data in the frame of reference of self versus other, and again the difference in the two candidate regression slopes survived formal statistical comparison (paired t[18] = 3.1, z = 2.74, p < 0.01; Figure 2C).

**Figure 3:**
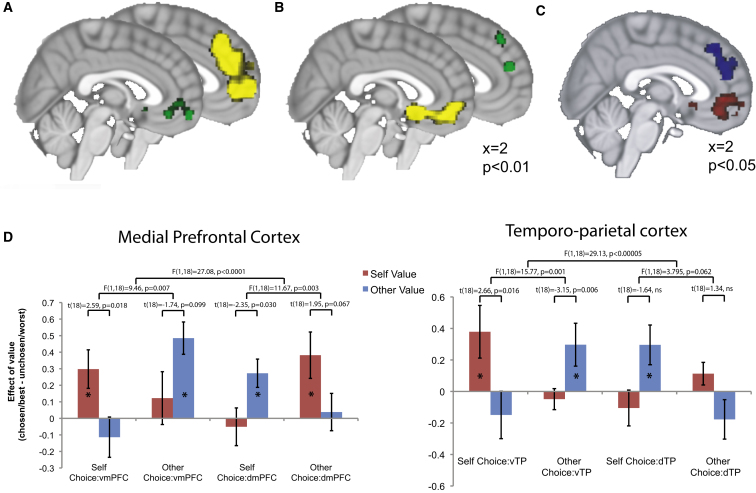
Neural Correlates of Value Difference during Delegated Intertemporal Choice (A) Activity while subjects choose for themselves. Green: Activity associated with subject’s chosen minus unchosen value difference. Yellow: Activity associated with partner’s preferred minus nonpreferred value difference. (B) Activity while the subject chooses for their partner. Green: Activity associated with subject’s preferred minus nonpreferred value difference. Yellow: Activity associated with the partner’s values in the frame of reference of the choices made by the subject on behalf of the partner (chosen value minus unchosen value). (C) Voxels showing a conjunction of the contrasts shown in green in (A) and yellow in (B) (executed value difference signals) are shown in red. Voxels showing a conjunction of contrasts shown in yellow in (A) and green in (B) (modeled value difference signals) are shown in blue. Individual maps are thresholded at p < 0.05 before the conjunction analysis. (D) Formal test that brain regions exchange agents between choice conditions in medial prefrontal and temporoparietal cortices. In each case, data are extracted from clusters defined by the opposite condition, ensuring no selection bias is present (see text and Supplemental Experimental Procedures). Bars show average effects of value (chosen/best – unchosen/worst) across subjects. Error bars show the standard error of the mean. All p values are two tailed, and refer to interaction effects. Significant effects of each bar against 0 (p < 0.05 two tailed) are marked with stars. See Table S1 and Figure S3.

These data identify a dorsal-ventral functional organization of the medial PFC that does not follow a frame of reference of self versus other, but instead is tied to a frame of reference of executed versus modeled choices. To explore the data that underlies this functional gradient, we looked at activity that correlated with subjective preference-related activity separately under each choice condition (choice for self or for other). In blocks where subjects chose on behalf of themselves, activity in vmPFC correlated with the difference between the subject’s valuation (discounted by their own discount rate) for the chosen and unchosen options (Figure 3A), thus reflecting their personal choice preferences (Montreal Neurological Institute [MNI] atlas 12, 53, −11, t = 3.31, z = 2.76). Simultaneously, dmPFC activity also exhibited a value difference correlate but here values were discounted according to the partner’s discount rate, and the relevant value difference was between the partner’s preferred and nonpreferred choices (MNI 3, 41, 25, t = 5.00, z = 3.75). Hence in self-choice trials, despite the fact that the partner’s valuation bore no relevance to the task, dmPFC activity nevertheless reflected the experimental subject’s estimate of their partner’s preferences.

As predicted by the gradient analysis, we observed a dramatically different pattern of activity during the delegated choice condition (Figure 3B). When subjects now made choices on behalf of their partner, these regions precisely swapped agents, such that the vmPFC now maintained an estimate of the partner’s values, expressed in the frame of reference of the choices made on behalf of the partner (MNI −6, 23, −11, t = 7.36, z = 4.94). Conversely, the dmPFC now reflected the subject’s own values, signed according to the choices that the subject themselves would have preferred in the same context (MNI 0, 50, 19, t = 4.01, z = 3.34). Accordingly, when we searched for regions that contained a conjunction of voxels responding to both types of executed, or choice-relevant, value differences (p < 0.05) we recovered a signal in vmPFC. At the same threshold, a region within dmPFC contained voxels representing modeled, or choice-irrelevant, value difference, be it those of the subject or those of the partner (Figure 3C).

The data presented in Figures 3A and 3B are not multiple comparison corrected and therefore do not constitute formal tests. We present these data to illustrate the effects in the individual conditions that underlie the formal tests of interaction. In order to provide a formal test that the regions switched agents between conditions, we designed a test that selected peaks exclusively from one choice condition and extracted data from these peaks in the alternative choice condition. Thus, peaks isolated from self-choice trials were used to assess data in other-choice trials, and vice versa. This test obviates questions of multiple comparisons, as peaks are selected from one set of data and tested in the independent alternative data set. From within the value-coding region shown in Figure 2A, we selected the peaks that correlated maximally with “self value-difference relative to other value-difference” and with “other value-difference relative to self value-difference” in each of self-choice and other-choice conditions. As predicted by the gradient analysis (Figure 2), this resulted in two peaks at the ventral extreme of the rmPFC (peak MNI −12, 26, −11, t = 3.57, z = 3.06 for self choices; peak MNI −3, 17, −8, t = 4.10, z = 3.40 for other choices) and two peaks at the dorsal extreme (peak MNI 3, 44, 25, t = 4.35, z = 3.55 for self choices; peak MNI 9, 38, 43, t = 5.06, z = 3.94 for other choices) in each condition. We therefore labeled these peaks vmPFC and dmPFC. We then extracted data from these peaks in the alternative condition. This allowed us to test several predictions that the regions switched agents between conditions, as detailed statistically in Figure 3D. In brief, vmPFC showed significant effects of self values, but not other values, during self-choice, and other values, but not self values, during other-choice. The interaction within vmPFC demonstrated that vmPFC switched its value coding. dmPFC showed significant effects of other values, but not self values, during self choice, and self values, but not other values, during other choice. Again, the interaction within dmPFC demonstrated a switched coding pattern. Finally, the formal three-way interaction between brain region, value-scheme and choice condition demonstrated that the two regions switched their coding in opposite fashions, and hence exchanged agents. Specifically, vmPFC always represented the values relevant for choice, while dmPFC always tracked the values irrelevant for choice (Figure 3D).

As temporoparietal cortex had exhibited a similar gradient to rmPFC, we also subjected this region to the test described above. That is, we tested whether neighboring subregions of temporoparietal cortex exchanged agents in the different choice conditions. Again, within a mask defined by the average value effect over both agents, we applied the same procedure in which peaks were selected from one choice condition, and data extracted from the other (Supplemental Experimental Procedures). This analysis revealed that, as in the medial prefrontal cortex, dorsal and ventral extremes of temporoparietal cortex exchanged agents between conditions. This was true whether data were averaged across hemispheres (Figure 3D) or tested independently in each hemisphere (Figure S3A).

## Discussion

Understanding the values and predicting the actions of other individuals is important for all social animals. In humans, social factors impinge on almost every decision that we make. Here, we show that when we make a value-dependent choice, other people’s values and preferences are represented in regionally distinct patterns of neural activity. Notably, the values of other people were identified with the same computational regressor (value difference) used to identify personal subjective values in imaging and single unit physiology studies (Basten et al., 2010; Boorman et al., 2009; Cai et al., 2011; FitzGerald et al., 2009), suggesting that similarities exist in the neural computations underlying self and other valuation. However, it was not the case that value computations for self and other were constrained to particular brain regions. Instead, the two representations swapped locations, both in the prefrontal cortex and in the temporoparietal cortex, depending on which valuation was relevant to the expression of a current choice.

The two prefrontal brain regions that form the central focus of our study have been extensively studied in neuroeconomics and social neuroscience. The vmPFC is a region that lies on the boundary of the pregenual cingulate cortex (areas 32,25), the orbitofrontal cortex (area 14) and the medial polar cortex (medial area 10). It is a region commonly implicated in stimulus valuation (Hare et al., 2011; Plassmann et al., 2007) and goal-directed choice (Basten et al., 2010; Hunt et al., 2012; Wunderlich et al., 2010, 2012). The rostral dmPFC lies close to the dorsal boundary of medial area 10, where it meets medial area 9. This region is not often highlighted in neuroeconomic studies of value outside the social domain, but is repeatedly activated in tasks that require subjects to attribute intention to other agents (Behrens et al., 2008, 2009; Frith and Wolpert, 2004; Hampton et al., 2008; Yoshida et al., 2010). While these activations have consistently occurred at the same anatomical locations in the human brain, the precise functional role of the region has been hard to decipher, partly as it is has not been clear that a homologous brain region exists in any nonhuman species (although see Sallet et al., 2011). It is notable that this region is both functionally and anatomically distinct from a more caudal region in the dmPFC at the boundary of presupplementary motor area, medial area 9, and the dorsal anterior cingulate cortex. This latter region is commonly implicated in valuation and choice, with opposing coding to vmPFC (Hare et al., 2011; Kolling et al., 2012; Wunderlich et al., 2009). Indeed, when we test the negative (i.e., unchosen minus chosen) contrast of executed value difference in our study, it is precisely this more caudal region that is revealed (Supplemental Experimental Procedures, Figure S3B).

Our data suggest that the functional organization in medial prefrontal cortex does not align to the frame of reference of the individual. Instead activity in vmPFC reflects a choice preference that is executed and rostral dmPFC a choice preference that is modeled. Thus, dorsal regions of rostral mPFC contain value representations in the frame of reference of a modeled (as opposed to an actuated) preference irrespective of whether this applies to another’s or to one’s own likely actions and goals. Likewise, ventral mPFC contains a representation of value in the frame of reference of an executed choice, even if this executed choice reflects one’s own or another’s preferences. It is notable that this is the case despite the fact that partners were explicitly selected to have opposing preferences (Jenkins et al., 2008).

While other-regarding activity has previously been observed in the vmPFC (Cooper et al., 2010; Hare et al., 2010), it has often been assumed that this is because the subject finds altruistic acts intrinsically rewarding (Fehr and Camerer, 2007). Indeed, it has been suggested that the valuation system in the vmPFC represents automated processing of subjective value (Kable and Glimcher, 2009; Lebreton et al., 2009; Rangel and Hare, 2010). However, this explanation cannot account for the current data where, during delegated choice, vmPFC reflects the preferences of the partner, correlating with the difference between the partner’s chosen and unchosen values, and not with the subject’s own choice-irrelevant preferences (which are instead tracked in dmPFC). Hence, in our task vmPFC activity reflects the selection of executed choices (Boorman et al., 2009; FitzGerald et al., 2009; Noonan et al., 2010), irrespective of whether these are in line with one’s own valuation.

Previous studies have highlighted mPFC’s role in understanding the intentions of other agents, so-called theory of mind (Amodio and Frith, 2006; Saxe, 2006), but attribute this function exclusively to dmPFC. More recently, computational accounts have prescribed precise computational functions to this dmPFC activity during social learning (Behrens et al., 2009; Behrens et al., 2008; Hampton et al., 2008). In the current study, we identify a signal in rostral dmPFC that reflects the values and preferences of another individual (here temporally discounted at a rate specific to the individual), even when they are not directly relevant to the task at hand. Critically, we also show this activity is not confined to simulating the actions of other individuals. When subjects are making value-based actions that they would not normally take (when acting on behalf of another person), their own values and preferred choices are represented in this same region of dmPFC. While the simplest interpretation of this effect is that the region is simulating one’s own, currently irrelevant, preferences an alternative possibility is that the activity is projecting one’s own values into the mind of the partner, while simulating the partner’s choices. In essence, estimating the extent to which my own values would influence my partner, if they were making the choice. This iterated reasoning would be consistent with previous suggestions that the dmPFC is implicated in such higher order belief inference (Yoshida et al., 2010).

However, while it may seem counterintuitive for neural processes to be dedicated to computing values and choices that do not pertain directly to current goals, such a process is likely to have importance in many cognitive functions outside social cognition. For example, optimal decision making may rely on the ability to model one’s own likely behavior in the context of future choices that ensue after an immediate action. Our observation that the exact same computational signals can be measured for oneself, and for a confederate, in both vmPFC and dmPFC offers evidence for the idea that self-referential processing and mentalising about others share common neural mechanisms (Amodio and Frith, 2006; Buckner and Carroll, 2007; Mitchell, 2009).

## Experimental Procedures

### Subject Recruitment and Task Optimization

For a detailed description of the subject screening, and the task optimization, see the Supplemental Information. In brief, we simulated 10,000 sets of 100 choices between a larger-later and smaller-sooner prizes, and selected the choice-pair set that led to the most efficient estimate of individual discount rates. We then screened 87 participants with these choices and selected the 20 subjects with the ten highest and ten lowest discount rates to form our participant pairs. Last, we simulated a further 10,000 sets of 120 choices for fMRI scanning that would minimize the correlations between predicted signals of the two players. We excluded one participant who was unable to replicate their partner’s choices in the scanner (30% difference between this subject’s choices and their partner’s actual choices by the end of the trial-and-error learning). This study was approved by the University College London Research Ethics Committee.

### FMRI

We acquired fMRI data using standard procedures (see Supplemental Experimental Procedures) designed to minimize susceptibility related artifacts in the ventral prefrontal cortex. After standard preprocessing (see Supplemental Information), we analyzed the data using a general linear model with the following regressors. In each condition (choose for self or other), we included a regressor defining the main effect of condition, and four parametric regressors reflecting the chosen and unchosen values for each party. For the currently relevant party, these were sorted according to the choices actually made. For the currently irrelevant party, they were sorted according to the choices that would have been made (i.e., the “chosen value” was always greater than the “unchosen value”). We then performed (1 −1) contrasts between each pair of chosen and unchosen values, to give the effects of value difference.

#### fMRI Statistics and Thresholding

The data presented in Figure 2 comprise formal statistical tests of execution versus modeling. In Figures 2A and 2B, clusters are thresholded at t > 3 and cluster corrected for the whole brain at p < 0.01. The tests in Figure 2C are not performed voxel-by-voxel but rather one for each potential gradient. A regression is performed in each subject with an indicator of dorsal-ventral position (1 to 5) as the independent variable, and the relevant BOLD contrast at each point as the dependent variable. A random effects t test is then performed on these gradients across the group. For the difference of gradients test, this is replaced by a paired t test reflecting the difference between gradients for executed-modeled and gradients for self-other. While these results would survive Bonferroni correction across several brain regions, we only in fact performed the spatial gradient analyses on axes within mPFC and TPC.

The data presented in Figure 3D also present a formal statistical test of execution versus modeling, in that they test whether the regions switch roles between conditions. The data shown in Figure 3D show value-related peaks in vmPFC and dmPFC selected from one choice condition and used to test the direction of value correlations in the alternative choice condition, therefore obviating questions of multiple comparisons.

The data presented in Figures 3A and 3B are shown so that the effects that underlie the statistical tests in the manuscript can be easily understood. They are figurative and therefore not corrected for multiple comparisons. Nevertheless, all shown clusters have peaks at p < 0.002 uncorrected.

## Supplemental Information

Document S1. Figures S1–S3, Tables S1–S3, and Supplemental Experimental Procedures
