# An Agent Independent Axis for Executed and Modeled Choice in Medial Prefrontal Cortex

**DOI:** 10.1016/j.neuron.2013.07.030

**Published:** 2013-08-07

**Authors:** Antoinette Nicolle, Miriam C. Klein-Flügge, Laurence T. Hunt, Ivo Vlaev, Raymond J. Dolan, Timothy E.J. Behrens

(Neuron *75*, 1114–1121; September 20, 2012)

As the result of a journal error during the production process, an incorrect version of the Supplemental Information was previously published with this article online. We have now corrected the online Supplemental Information and apologize for the error.

