# Supplementary material for: An Agent Independent Axis for Executed and Modeled Choice in Medial Prefrontal Cortex
Source: Neuron. 2012 Sep 20;75(6):1114–21. doi: 10.1016/j.neuron.2012.07.023 (PMC3458212; doi:10.1016/j.neuron.2012.07.023)
Supplement: Document S1. Figures S1–S3, Tables S1–S3, and Supplemental Experimental Procedures [file mmc1.pdf]

Neuron, volume *75*

Supplemental Information

An Agent Independent Axis or Executed and Modeled Choice in Medial Prefrontal Cortex

Antoinette Nicolle, Miriam C. Klein-Flügge, Laurence T. Hunt, Ivo Vlaev, Raymond J. Dolan, and Timothy E.J. Behrens

Inventory of supplemental materials.

Figure S1: Optimisation of task choices. Related to main figure 1.

Table S1: Four example trials illustrating decorrelation approach. Related to main figure 1.

Figure S2: PFC gradients in individual subjects, and TPJ gradient across the group. Realted to main figure 2.

Table S2. While brain clusters for contrast in figure 2. Related to main figure 2.

Table S3. Whole brain clusters for contrast in figure 3a,b. Related to figure 3.

Figure S3. Main figure 3D broken down into hemispeheres, and inverse contrasts of main figure 3a,b. Related to figure 3.

Supplemental Information

Supplemental Experimental Procedures

Optimisation of the choice pairs used in the task

The goal of our experiment meant that it was necessary to optimise the choice pairs that we used both in the screening questionnaire and in the main fMRI task.

In the pre-screening it was important to optimise choices such to give the most efficient estimate of potential subjects' discount rates. To optimise these choices, we generated a random 100 choice pairs, each comprising one smaller, sooner reward and one larger, more delayed reward, but with the magnitudes and delays varying across the pairs. We then computed the decisions predicted by simulated subjects whose discount rates ranged from 0 to 1. When plotted (figure S1), the closer this graph is to the diagonal, the better different discount rates are reflected in different subject's choice preferences, and therefore the lower the error introduced by the model estimation process. We generated 10,000 such choice sets and chose the set whose curve was closest to the diagonal (in terms of enclosed area). Figure S1 plots the predicted choices from this choice set.

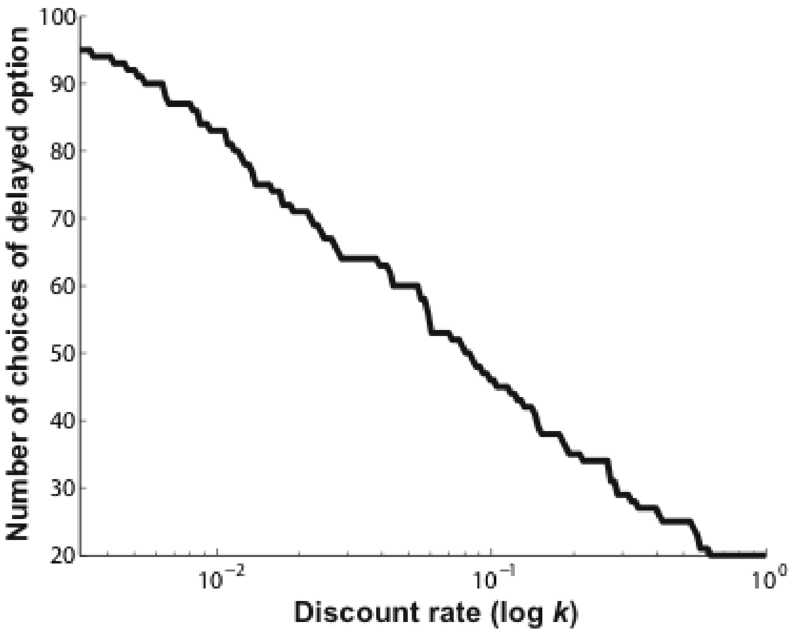

Figure S1, related to figure 1

The choice set was optimised, such that different discount rates (k) would be reflected in different choices.

In the fMRI task, the choice set was optimised to minimise the correlation between the fMRI regressors of individuals with sufficiently divergent discount rates – we expected that the mPFC BOLD signals would correlate with value difference (Boorman, Behrens, Woolrich, & Rushworth, 2009; FitzGerald, Seymour, & Dolan, 2009) and so we ensured that the value difference (chosen minus unchosen values) would decorrelate across partners selected to have different discount rates. However, we took the precaution to also ensure that the chosen value signals would also decorrelate across the two partners, as there have been some reports of vmPFC correlates of chosen value (Wunderlich, Rangel, & O'Doherty, 2010).

To optimise the choice pairs for these purposes, we used a procedure similar to above. Here, each choice set consisted of 120 pairs. Again, we generated 10,000 random examples of such choice pairs. Now, however, we computed the value regressors for 100 simulated individuals

with discount rates ranging from 0 to 1. We computed the cross-correlation matrix between the value regressors for each simulated subject (figure 1b), and selected the choice-pair set that generated the lowest average correlation across all simulated subjects. In this choice set, magnitudes ranged between £0.92 and £34.56 for sooner options, with their delays ranging from 0 to 6 weeks. For the more delayed options of each pair, the magnitudes ranged from £5.79 to £74.38, and the length of delays from 1 to 75 weeks, since these provided the lowest correlations in self and other choices and values. This procedure ensured we could subsequently select partners whose signals (unique to their personal discount rate) could be dissociated in the value-related BOLD signals of interest (see below).

It may seem counter-intuitive that two players with opposing preferences can have decorrelated value functions. However, it turns out this is possible if they are faced with carefully selected choices. For example, consider the four trials shown in the following Table S1. In these 4 trials, the low discounter has value differences of (5,100,100,0); the high discounter has value differences of (100,100,5,5). The correlation between these two vectors is 0, despite the fact that the opposite choice was made on three of the four trials.

Table S1, related to Figure 1.

Choice A	105 today	100 today	30 today	20 today
Choice B	110 6 months	0 today	130 two months	25 tomorrow
Low discounter A value	105	100	30	20
Low discounter B value	110	0	130	25
High discounter A value	105	100	30	20
High discounter B value	5	0	25	15
**Low discounter value difference**	**5**	**100**	**100**	**5**
**High discounter value difference**	**100**	**100**	**5**	**5**

Training and performance on delegated choice task

Prior to scanning, partners completed a trial-and-error learning task in a context where each could learn their partner's preferences by reviewing the choice pairs used in the screening questionnaire and selecting the options they thought might have been previously chosen by their partner. Trial-by-trial feedback informed participants whether or not they had correctly estimated their partner's choice. Participants were effective in learning to replicate their partner's choices as their total number of smaller-sooner choices differed, on average, by only 4.5% (figure 1d).

Then, during fMRI scanning, participants were presented with a new set of intertemporal choices in blocks of 40 trials. In each block they made choices either on behalf of themselves, or their partner. At the end of the experiment two of their actual choices would be realised – one prize randomly selected from the self-regarding blocks would go to the subject, and one from the other-regarding blocks to the partner. Again in this experimental context subjects were reliably able to replicate their partner's behaviour (8.24% difference between the partner's choices and the subject's estimation of the partner's choices (fig 1d)).

The temporal discounting model

Each pre-screened participant's unique discount rate was estimated by fitting a discount function to their 100 questionnaire choices, whereby the discounted value of an option (*Vd*) varies hyperbolically as a function of reward magnitude (*M*) and delay (*D*). The impact of delay depends on the participant's unique discount rate (*k*).

$$Vd = \frac{M}{1 + kD}$$

Given a subjective (i.e. temporally discounted) value associated with each of the two options in the pair, the associated probability of making each choice is estimated through the following logit transform where $\beta > 0$ and determines the randomness of the decision (with larger numbers indicating more random choice):

$$p_i = \frac{\exp(\beta V d_i)}{\sum_{j=1,2} \exp(\beta V d_j)}$$

This is a standard stochastic decision rule that calculates the probability of taking one of two actions according to their relative subjective values.

This model was fit to participants' choices in the pre-screen questionnaire, optimising the model's free parameters (k) and (β) to maximise the likelihood of the choices given the parameters. This was realised through standard Matlab functions. We used Bayesian information criterion (BIC) to compare model performance between self and other choice conditions (Schwarz, 1978).

Model fitting results

During pre-screening, the mean fitted discount rate was 0.02 for the low discounters selected for the main experiment (se=0.004) and 0.75 for the high discounters (se=0.12).

During scanning, the model fitted equally well when choosing for self (BIC 75.36 (se=6.13)) and other (BIC 76.31 (se=4.90)). For low discounters, the mean fitted discount rate was 0.03 when choosing for self (se=0.01) and 0.80 when choosing for their partner (se=0.11). For

high discounters, the mean fitted discount rate was 0.63 when choosing for self (se=0.15) and 0.02 when choosing for their partner (se=0.01).

The value regressors used for analysing the fMRI data were computed using the hyperbolic discount function and the participant's own unique discount rate (for self-relevant values) and their partner's discount rate (for other-relevant values). For our study, it was vital that these regressors were decorrelated with each other, as would be predicted by the divergent discount rates of our participant pairs. This was indeed the case. There were no significant correlations between the self value (chosen minus unchosen) and other value (would have been chosen minus would have been unchosen) regressors, both when choosing for self (mean $R = -0.094$, $p=0.36$) and when choosing for other (mean $R = -0.134$, $p=0.20$).

fMRI data acquisition

We scanned participants in a 3T Allegra head scanner (Siemens, Erlangen, Germany) operated with its standard head transmit-receive coil. The manufacturer's standard automatic 3D-shim procedure was performed at the beginning of each experiment. Participants were scanned with a single-shot gradient-echo EPI sequence, optimized to reduce BOLD sensitivity losses in the orbitofrontal cortex due to susceptibility artifacts (Weiskopf, Hutton, Josephs, & Deichmann, 2006). Imaging parameters were as follows: 48 oblique transverse slices tilted by 45°, slice thickness = 2mm with a 1mm gap between slices, repetition time TR = 2.88s, $\alpha = 90°$, echo time TE = 30ms, BWPE = 42 Hz/pixel, negative phase-encoding gradient polarity, field of view = 192×216 mm^2, 3×3 mm in-plane resolution, matrix size 64×72, fat suppression, z-shim gradient pre-pulse moment = -1.4 mT/m×ms. EPI data acquisition was monitored on-line using a real-time reconstruction and quality assurance system (Weiskopf et al., 2007). We acquired fieldmaps for each subject at the start of

scanning (Siemens standard double echo gradient echo fieldmap sequence, echo time = 12.46 ms, TR = 10.2 ms, matrix size = 64×64, 64 slices covering the whole head, voxel size=3×3×3mm). These allowed calculation of static geometric distortions caused by susceptibility-induced field inhomogeneities, which were used to correct EPI images for both these static distortions and any changes in these distortions due to head motion (Andersson, Hutton, Ashburner, Turner, & Friston, 2001; Hutton et al., 2002). We also recorded heart rate with a pulse oximeter, along with respiratory phase and volume using a breathing belt, which were used to correct for physiological noise during data analysis. At the end of the scanning session, we acquired a T1-weighted anatomical scan for each participant using a Modified Driven Equilibrium Fourier Transform (MDEFT) sequence (Uğurbil et al., 1993), with optimised parameters as described in the literature (Deichmann, Schwarzbauer, & Turner, 2004). For each participant, 176 sagittal partitions were acquired with an image matrix of 256×224 (Read × Phase).

fMRI data analysis

Image pre-processing and data analysis were implemented using Statistical Parametric Mapping software in Matlab R2010b (SPM8; Wellcome Trust Centre for Neuroimaging, at UCL). After discarding the first 6 volumes of each session, to allow for T1 equilibration, EPI images were corrected for geometric distortions caused by susceptibility-induced field inhomogeneities. Fieldmaps were processed for each participant using the FieldMap toolbox (Hutton, Deichmann, Turner, & Andersson, 2004). The EPI images were then realigned and unwarped (Andersson et al., 2001), and each participant's structural image was then co-registered to the mean of the motion-corrected functional images using a 12-parameter affine transformation, and segmented according to the standard procedure in SPM8 (Ashburner &

Friston, 2005). The spatial normalization parameters resulting from the previous step were then applied to the functional images to allow for inter-subject analysis, and finally these images were smoothed using an 8mm FWHM Gaussian kernel, in accord with the standard SPM approach.

For each participant, we constructed an event-related general linear model, including regressors for our value signals of interest (see main text methods). One participant was removed from analysis, after their choices during scanning indicated especially low accuracy in replicating their partner's preferences (30% difference). Trials in which participants chose for self and for other were modelled on separate regressors, and each was parametrically modulated by the mean-corrected self and other values. These value regressors were computed trial-by-trial based on the self and other specific discount rates, with increasing numbers reflecting the parametric effect of increasing value. Values for the options chosen and unchosen (or what would have been chosen or unchosen when the values were irrelevant for choice) were computed and modelled separately so that their difference could be taken. Values in each regressor were mean corrected and normalised to unit standard deviation. Onsets were set at the start of the choice phase (i.e. when the two choice options appear on the screen), modelled with a duration of the length of the particular trial's RT (or up to 4 seconds, whichever was sooner), and convolved with the standard canonical haemodynamic response function in SPM8. Button presses were modelled as a regressor of no interest parametrically modulated by RT. Head motion parameters defined by the realignment procedure were entered as 6 regressors of no interest, along with 17 additional regressors of cardiac phase (10 regressors), respiratory phase (6 regressors) and respiratory volume (1 regressor). For details of thresholding and statistical testing, see main text.

Breakdown of peaks selected for figure 3D temporo-parietal cortex results

As shown in figure 2b and figure S2e, a dorso-ventral gradient existed bilaterally in temporo-parietal cortex along the axis of executed vs. modelled value differences. We therefore subjected these regions to a formal test that the dorsal and ventral portions of temporo-parietal cortex (TP) switched agents between conditions. Data were extracted from the following value-related peaks (in MNI space) in one choice condition in order to test the direction of value correlations in the alternative choice condition, therefore obviating questions of multiple comparisons.

Right hemisphere:

ventral TP – selected from other choice, other minus self value difference, and used for extracting data from the self choice condition = -42, -55, 19 (t=4.54. z=3.66)

ventral TP – selected from self choice, self minus other value difference, and used for extracting data from the other choice condition = -63,-61,16 (t=3.98, z=3.33)

dorsal TP – selected from other choice, self minus other value difference, and used for extracting data from the self choice condition = -42,-52,43 (t=2.68, z=2.43)

dorsal TP – selected from self choice, other minus self value difference, and used for extracting data from the other choice condition = -48,-37,46 (t=2.40, z=2.21)

Left hemisphere:

ventral TP – selected from other choice, other minus self value difference, and used for extracting data from the self choice condition = 51,-52,13 (t=3.51, z=3.02)

ventral TP – selected from self choice, self minus other value difference, and used for extracting data from the other choice condition = 54,-40,16 (t=3.91, z=3.28)

dorsal TP – selected from other choice, self minus other value difference, and used for extracting data from the self choice condition = 36,-67,43 (t=2.77, z=2.50)

dorsal TP – selected from self choice, other minus self value difference, and used for extracting data from the other choice condition = 51,-40,46 (t=3.41, z=2.95)

Supplemental Figures

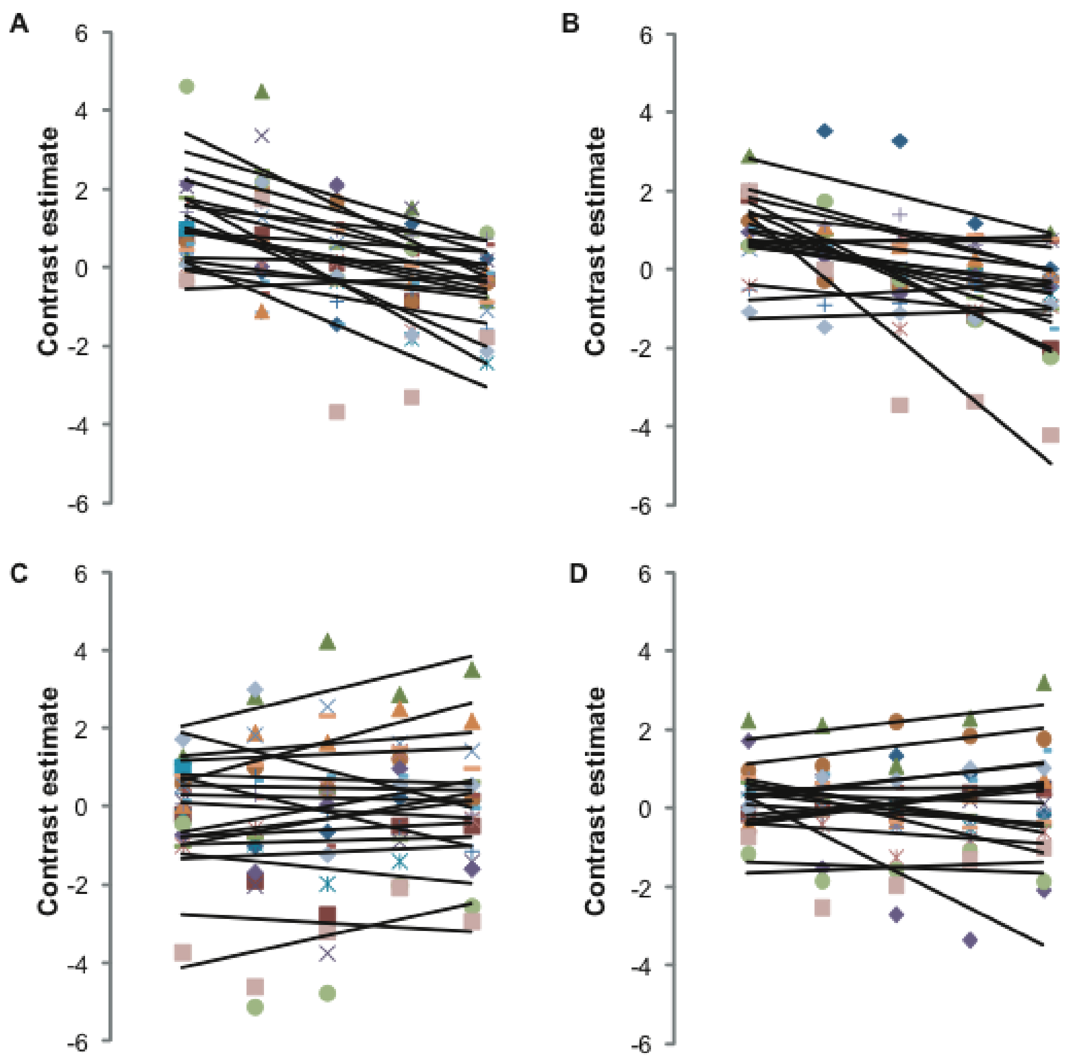

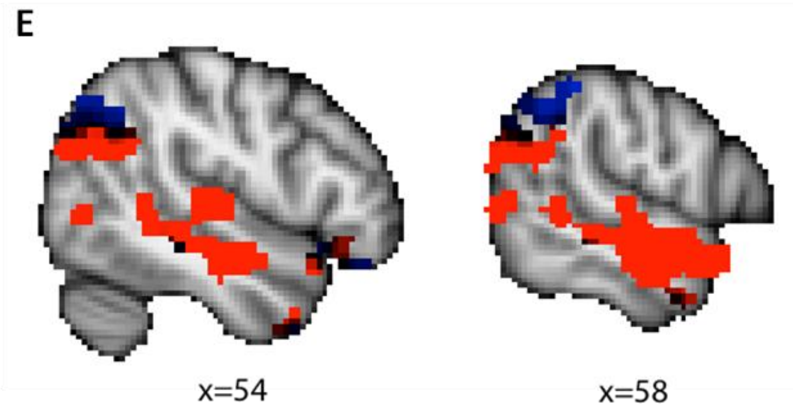

Figure S2, related to Figure 2.

A-D show the subject-by-subject gradients for executed minus modelled value in the mPFC (a) and TPC (b), while there is a lack of such a gradient for self minus other values in both the mPFC (c) and TPC (d). The x axis shows the gradient from most ventral (left) to most dorsal (right).

E shows sagittal slices through right temporo-parietal cortex. This is exactly as in Figure 2b, but showing the location of temporo-parietal cortex gradient on sagittal sections.

Table S2, related to Figure 2: Effects of executed vs. modelled value differences averaged across conditions (as shown in figure 2b).

Brain regions	MNI coordinates of local maxima	Voxel number at p<0.001 uncorrected	Voxel t score
**Executed Value (Chosen > Unchosen) > Modelled Value (Best > Worst)**			
Right middle temporal gyrus	54, -7, -11	715	7.90

Left Temporoparietal cortex (including ventral TPJ)	-39, -64, 22	364	7.59
Posterior Cingulate	-18, -40, 58	1179	7.14
Left temporal pole	-48, 11, -38	81	6.74
vmPFC	0, 14, -8	254	6.56
Left fusiform gyrus	-39, -16, -23	552	6.37
Left parahippocampal gyrus	-24, -37, -17	90	5.58
Right Temporoparietal cortex (including ventral TPJ)	60, -61, 4	409	5.57
**Modelled Value (Best > Worst) > Executed Value (Chosen > Unchosen)**			
Left dmPFC	-12, 47, 22	84	4.57
Right middle frontal gyrus	42, 20, 49	35	4.53
Right dorsal TPJ	51, -67, 37	32	4.32
Posterior Cingulate	3,- 40, 3	7	4.1

***Table S3, related to Figure 3**:* Whole-brain corrected activations associated with the simple effects of value (and value differences) illustrated in figures 3A and B. Results are only shown if whole-brain FWE corrected at $p < 0.05$, with a threshold of $p < 0.001$ voxel-level uncorrected and a cluster extent threshold of 20 voxels.

Brain regions	MNI coordinates of local maxima	Voxel number at p<0.001 uncorr	Voxel t score
**Self Chosen Value during Self Choice**			
Left Middle temporal	-60, -64, 22	171	6.09
Temporal lobe	42, -25, -8	140	6.09

Trend Right Middle temporal	63, -58, 13	66	5.72
**Self Unchosen Value during Self Choice**	No significant results		
**Self Value Chosen > Unchosen during Self choice**			
Trend Angular Gyrus	-54, -55, 25	89	5.50
**Self Value Unchosen > Chosen during Self choice**	No significant results		
**Other Best Value during Self Choice**	No significant results		
**Other Worst Value during Self Choice**	No significant results		
**Other Value Best > Worst during Self Choice**			
Trend Middle frontal	24, 56, 28	62	5.93
Anterior cingulate	3, 41, 25	79	5.00
**Other Value Worst > Best during Self Choice**	No significant results		
**Other Chosen Value during Other Choice**			
Fusiform gyrus	47, -25, -17	410	7.55
Middle temporal	-63, -55, 10	644	7.00
Lingual gyrus	-15, -64, -5	596	6.77
Postcentral gyrus	39, -40, 64	1426	6.55
Medial orbital	-6, 17, -14	349	5.89
Inferior temporal	51, -46, -26	88	5.83
**Other Unchosen Value during Other Choice**	No significant results		
**Other Value Chosen > Unchosen during Other Choice**			
Postcentral gyrus	27, -43, 64	1498	9.29

Medial orbital	-6, 23, -11	221	7.36
Fusiform gyrus	-27, -37, -17	75	7.09
Superior temporal	57, -1, -8	1226	6.91
Middle temporal	-48, -1, -20	235	6.30
Parahippocampal	-21, -10, -29	84	6.04
**Other Value Unchosen > Chosen during Other Choice**			
Insula	-30, 26, -2	51	7.32
**Self Best Value during Other Choice**	No significant results		
**Self Worst Value during Other Choice**	No significant results		
**Self Value Best > Worst during Other Choice**	No significant results		
**Self Value Worst > Best during Other Choice**	No significant results		

Supplemental Analyses

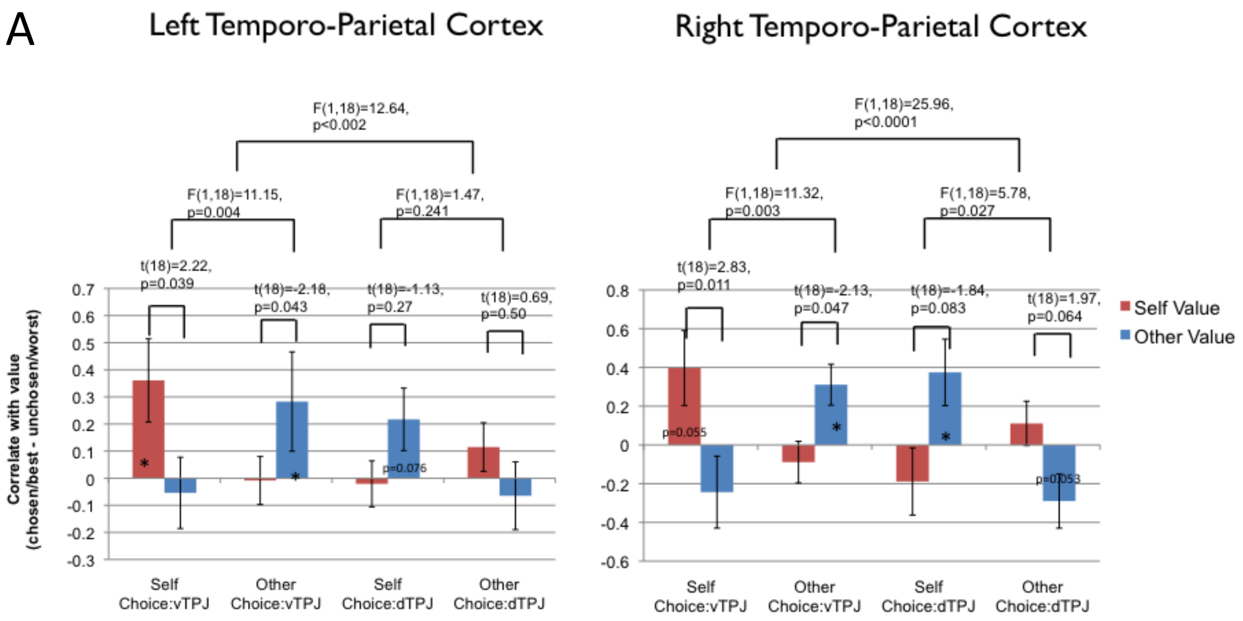

B

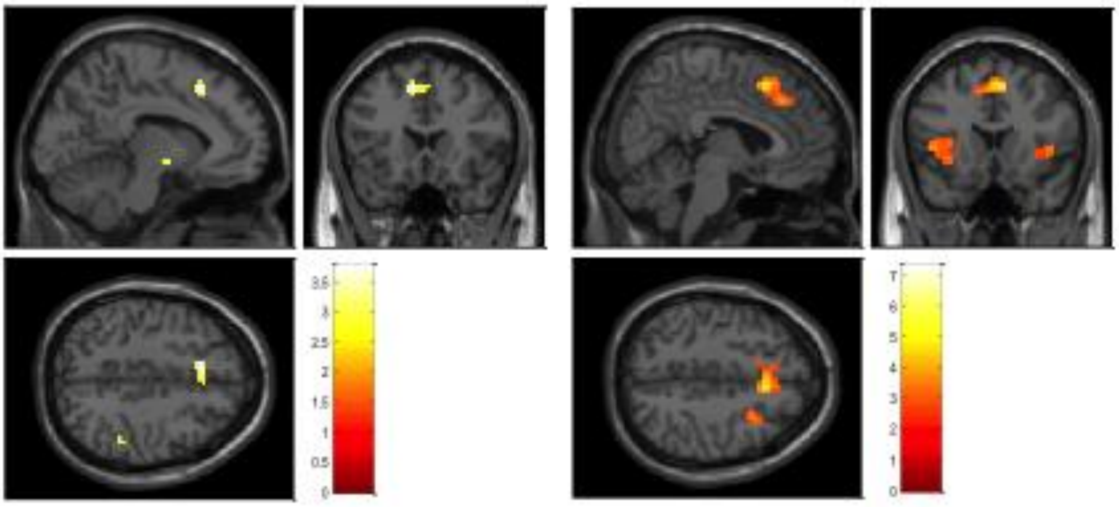

Figure S3, related to Figure 3.

A: Formal test that temporo-parietal cortices exchange agents between choice conditions, as in figure 3d, but data shown separately for both hemispheres.

B: A region of dorsal ACC/pre-SMA was found to be correlated with unchosen (minus chosen) executed values, both in the case of self value during choices for self (left figure, MNI peak -9, 20, 46, t=3.76, z=3.19) and other value during choices for other (right figure, MNI peak 6, 17, 49, t=5.26, z=4.04). These regions were caudal to the modelled value difference signal reported in the main paper.

This effect of unchosen value is notable because the region is adjacent to the rostral dmPFC that we focus on in the study, but it has a very different coding pattern (it codes for executed rather than modelled value difference, and it does so negatively rather than positively). This region lies on the boundary of the Anterior cingulate cortex and the pre-supplementary motor area. It is different both anatomically and functionally from the more rostral dmPFC region that we have focused on in this study. This ACC/pre-SMA region is commonly activated in

conflict studies (Ridderinkhof, Ullsperger, Crone, & Nieuwenhuis, 2004); and in decision-making studies, where it often correlates inversely with value difference (Hare, Schultz, Camerer, O'Doherty, & Rangel, 2011; Wunderlich, Rangel, & O'Doherty, 2009). The region that we have focused on for this study is rostral to this ACC/pre-SMA region. It is on the boundary of medial area 9 and medial polar area 10. Whilst its function is much less well defined, it is predominantly activated in studies of theory of mind (Frith & Frith, 2006; Hampton, Bossaerts, & O'Doherty, 2008), social cognition (Behrens, Hunt, & Rushworth, 2009; Behrens, Hunt, Woolrich, & Rushworth, 2008) and goal-directed planning (Yoshida & Ishii, 2006). Indeed, in figure 2 of the main text, we deliberately focus only on a region of interest that correlates positively on average with value difference. That is, we are deliberately excluding from the analysis regions such as the ACC/preSMA that correlate negatively with value difference.

Removal of possible outlier participant pair

Any correlation between value difference regressors of the two confederates will reduce the sensitivity of the test by increasing the variance of the contrast estimate. One participant pair were slightly more anticorrelated than all others. They can be seen as an outlier on the correlation plot in Figure 1C. Indeed, this pair only exhibited a correlation in the region of -0.4, meaning that the two regressors shared only approximately 16% of their variance. However, to be sure that our effects were not being driven significantly by this pair, we re-ran our analyses after removing these two participants.

After removing this participant pair, the key tests of the gradient analysis within mPFC (as in Figure 2C) remained significant: Exec – mod gradient $t(16)=6.26$, $p<0.00005$. Self – other gradient $t(16)=-0.807$, $p>0.4$. Paired t-test $t(16)=5.76$, $p<0.00005$.

The key results of the switching analysis (as in Figure 3D) also remained significant after removing this outlier pair: 3-way interaction $F(1,16)=21.91$, $p<0.0001$, vmPFC 2way interaction = $F(1,16)=7.63$, $p=0.014$, dmPFC 2way interaction $F(1,16)=9.43$, $p=0.007$.

Supplemental References

Andersson, J. L. ., Hutton, C., Ashburner, J., Turner, R., & Friston, K. (2001). Modeling geometric deformations in EPI time series. *Neuroimage*, *13*(5), 903–919.

Ashburner, J., & Friston, K. (2005). Unified segmentation. *Neuroimage*, *26*(3), 839–851.

Behrens, T. E. J., Hunt, L. T., & Rushworth, M. F. S. (2009). The computation of social behavior. *Science*, *324*(5931), 1160–1164.

Behrens, T. E. J., Hunt, L. T., Woolrich, M. W., & Rushworth, M. F. S. (2008). Associative learning of social value. *Nature*, *456*(7219), 245–249.

Boorman, E. D., Behrens, T. E. J., Woolrich, M. W., & Rushworth, M. F. S. (2009). How green is the grass on the other side? Frontopolar cortex and the evidence in favor of alternative courses of action. *Neuron*, *62*(5), 733–743.

Deichmann, R., Schwarzbauer, C., & Turner, R. (2004). Optimisation of the 3D MDEFT sequence for anatomical brain imaging: technical implications at 1.5 and 3 T. *Neuroimage*, *21*(2), 757–767.

FitzGerald, T. H. B., Seymour, B., & Dolan, R. J. (2009). The role of human orbitofrontal cortex in value comparison for incommensurable objects. *The Journal of Neuroscience*, *29*(26), 8388.

Frith, C., & Frith, U. (2006). The neural basis of mentalizing. *Neuron*, *50*(4), 531–534.

Hampton, A. N., Bossaerts, P., & O'Doherty, J. P. (2008). Neural correlates of mentalizing-related computations during strategic interactions in humans. *Proceedings of the National Academy of Sciences*, *105*(18), 6741.

Hare, T. A., Schultz, W., Camerer, C. F., O'Doherty, J. P., & Rangel, A. (2011). Transformation of stimulus value signals into motor commands during simple choice. *Proceedings of the National Academy of Sciences*, *108*(44), 18120–18125.

Hutton, C., Bork, A., Josephs, O., Deichmann, R., Ashburner, J., & Turner, R. (2002). Image distortion correction in fMRI: a quantitative evaluation. *Neuroimage*, *16*(1), 217–240.

Hutton, C., Deichmann, R., Turner, R., & Andersson, J. L. R. (2004). Combined correction for geometric distortion and its interaction with head motion in fMRI. *Proceedings of ISMRM* (Vol. 12).

Ridderinkhof, K. R., Ullsperger, M., Crone, E. A., & Nieuwenhuis, S. (2004). The role of the medial frontal cortex in cognitive control. *Science's STKE*, *306*(5695), 443.

Schwarz, G. (1978). Estimating the dimension of a model. *The annals of statistics*, *6*(2), 461–464.

Uğurbil, K., Garwood, M., Ellermann, J., Hendrich, K., Hinke, R., Hu, X., Kim, S. G., et al. (1993). Imaging at high magnetic fields: initial experiences at 4 T. *Magnetic Resonance Quarterly*, *9*(4), 259.

Weiskopf, N., Hutton, C., Josephs, O., & Deichmann, R. (2006). Optimal EPI parameters for reduction of susceptibility-induced BOLD sensitivity losses: A whole-brain analysis at 3 T and 1.5 T. *NeuroImage*, *33*(2), 493–504. doi:10.1016/j.neuroimage.2006.07.029

Weiskopf, N., Sitaram, R., Josephs, O., Veit, R., Scharnowski, F., Goebel, R., Birbaumer, N., et al. (2007). Real-time functional magnetic resonance imaging: methods and applications. *Magnetic Resonance Imaging*, *25*(6), 989–1003.

Wunderlich, K., Rangel, A., & O'Doherty, J. P. (2009). Neural computations underlying action-based decision making in the human brain. *Proceedings of the National Academy of Sciences*, *106*(40), 17199–17204.

Wunderlich, K., Rangel, A., & O'Doherty, J. P. (2010). Economic choices can be made using only stimulus values. *Proceedings of the National Academy of Sciences*, *107*(34), 15005–15010.

Yoshida, W., & Ishii, S. (2006). Resolution of uncertainty in prefrontal cortex. *Neuron*, *50*(5), 781–789.
